# [Retracted] miRNA‑490‑3p promotes the metastatic progression of invasive ductal carcinoma

**DOI:** 10.3892/or.2024.8802

**Published:** 2024-08-26

**Authors:** Ning Lu, Mei Zhang, Lu Lu, Yan-Zhao Liu, Hai-Hong Zhang, Xiao-Dong Liu

Oncol Rep 45: 706–716, 2021; DOI: 10.3892/or.2020.7880

Following the publication of this paper, it was drawn to the Editor's attention by a concerned reader that certain of the western blotting data shown in Fig. 2D, the cell migration and invasion assay data in Fig. 3C, the mouse imaging pictures in Fig. 4C and D, and the H&E-stained images in Fig. 4E and F were strikingly similar to data appearing in different form in other articles written by different authors at different research institutes that had already been submitted or published elsewhere prior to the submission of this paper to *Oncology Reports.* Given that the abovementioned data had already apparently been submitted or published prior to the receipt of this paper at *Oncology Reports*, the Editor has decided that this paper should be retracted from the Journal. After having been in contact with the authors, they accepted the decision to retract the paper. The Editor apologizes to the readership for any inconvenience caused.

